# Biomechanical Effects of Plastic Heel Cup on Plantar Fasciitis Patients Evaluated by Ultrasound Shear Wave Elastography

**DOI:** 10.3390/jcm11082150

**Published:** 2022-04-12

**Authors:** Che-Yu Lin, Pei-Yu Chen, Shin-Han Wu, Yio-Wha Shau, Chung-Li Wang

**Affiliations:** 1 Institute of Applied Mechanics, College of Engineering, National Taiwan University, No. 1, Sec. 4, Roosevelt Road, Taipei City 10617, Taiwan; cheyu@ntu.edu.tw (C.-Y.L.); ywshau@gmail.com (Y.-W.S.); 2 Department of Orthopaedic Surgery, School of Medicine, College of Medicine, National Taiwan University, No. 1, Sec. 1, Ren-Ai Road, Taipei City 10051, Taiwan; f92548050@gmail.com; 3 Department of Orthopaedic Surgery, National Taiwan University Hospital, No. 7, Chung-Shan South Road, Taipei City 10043, Taiwan; 4 Department of Orthopaedic Surgery, Taitung Christian Hospital, No. 350, Kai-Feng Street, Taitung City 950405, Taiwan; shinhanjohn@gmail.com

**Keywords:** plantar heel pain, heel pad, foot and ankle, foot orthoses, ultrasound imaging

## Abstract

The plastic heel cup has been adopted to treat plantar heel problems for years. However, its mechanisms and biomechanical effects are yet to be fully understood. The purpose of this study was to investigate the effects of the plastic heel cup on the microchamber and macrochamber layers of the heel pad by comparing the stiffness (in terms of the shear wave speed) and thickness of these two layers with and without a plastic heel cup during static standing. Fifteen patients with unilateral plantar fasciitis were recruited. The shear wave speed and thickness of the microchamber and microchamber layers of each symptomatic heel pad during standing measured by ultrasound shear wave elastography were compared between conditions with and without a plastic heel cup. It was found that a plastic heel cup reduced the shear wave speed of the microchamber layer to 55.5% and increased its thickness to 137.5% compared with the condition without a plastic heel cup. For the microchamber layer, the shear wave speed was reduced to 89.7%, and thickness was increased to 113.6% compared with the condition without a plastic heel cup. The findings demonstrate that a plastic heel cup can help to reduce the stiffness and increase the thickness for both layers of the heel pad during standing, suggesting that the mechanism of a plastic heel cup, and its resulting biomechanical effect, is to reduce the internal stress of the heel pad by increasing its thickness through confinement.

## 1. Introduction

Plantar heel pain is considered a major public health problem and is one of the most common musculoskeletal complaints in elderly people, as well as in athletic populations [1,2]. It significantly limits daily life and sports activities and has a detrimental impact on health-related quality of life [1,3]. Since plantar heel pain is a serious problem in modern society, how to treat plantar heel pain quickly and efficiently has been a hot topic in medical research. There are numerous conservative approaches for treating plantar heel pain, including stretching, night splint, physical therapy, extracorporeal shockwave therapy, nonsteroidal anti-inflammatory drugs, and foot orthoses [4]. In order to optimize the treatment effect for plantar heel pain, there is an essential need to further investigate the underlying mechanism of each treatment approach.

**Figure 1:**
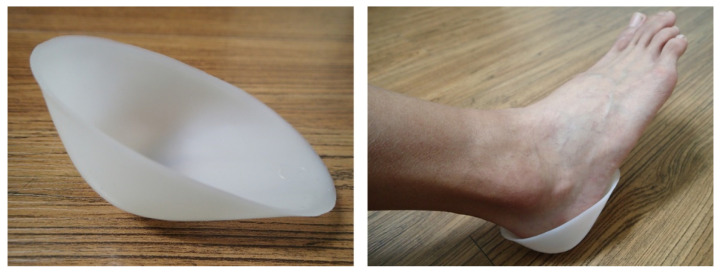
Illustration of plastic heel cup.

**Figure 2:**
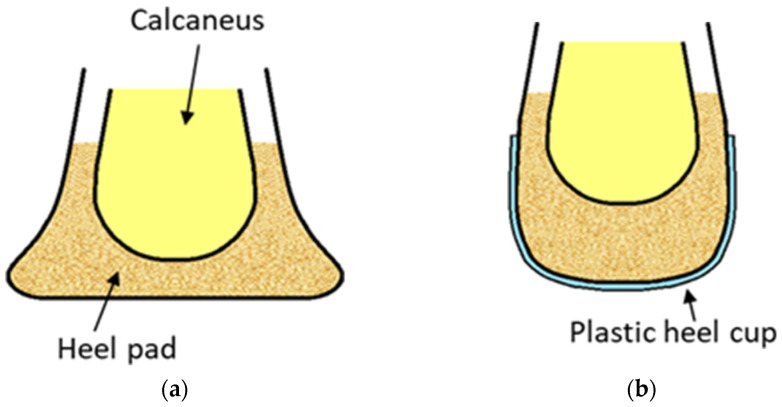
Illustration of the mechanism of the plastic heel cup. (**a**) The heel pad deforms significantly and becomes thinner under loading condition. (**b**) The plastic heel cup confines the heel, helping maintain the thickness of the heel pad.

The plastic heel cup (or called M-F heel protector in the commercial market), as shown in Figure 1, is a kind of orthotic device for treating plantar heel pain [5]. By inserting a plastic heel cup into the shoe around the heel, it encloses the soft tissues around the heel and provides additional confinement for the heel. Since originally developed for athletes suffering from heel bruises [5], the plastic heel cup has been adopted to relieve plantar heel problems in clinical practice for several decades, dating back at least to 1966 [6]. Plastic heel cups are inexpensive and effective in relieving plantar heel pain rapidly and have been recommended as one of the initial therapies for treating plantar heel pain [5]. It has been demonstrated that the mechanism of the plastic heel cup or similar devices is to increase the inherent shock absorption ability of the heel pad by preventing collapse of the heel pad through confinement (Figure 2). Plastic heel cups also can reduce plantar pressure and can reduce and change the pattern of ground reaction impact forces [7,8,9,10,11]. However, it is still unknown how the use of plastic heel cups can affect the internal stress of the heel pad under loading. The internal stress is a mechanical indicator of the magnitude of internal force intensity sustained by the tissue under loading that could be related to clinical symptoms [12,13,14]. It is also essential to separately investigate how the different layers of the heel pad, i.e., the microchamber layer (MIC) and macrochamber layer (MAC), respond mechanically to the use of a plastic heel cup, since these two layers have different functional roles [15,16,17,18,19,20,21].

Previously, it was a challenge to implement noninvasive evaluation of the internal stress of tissues in vivo. Thanks to the advancement of technology, ultrasound elastography is an emerging technique that could fulfill this need. Based on the fact that the shear wave speed is directly related to the stiffness of tissues, supersonic shear wave elastography (SWE) is a kind of ultrasound elastography that can quantitatively evaluate the stiffness of tissues by generating shear wave propagation within tissues through acoustic radiation force and then measuring the speed of shear wave propagation [22,23]. Previous studies have shown that the stiffness of tissues can be used as a metric for estimating the amount of internal stress sustained by tissues under loading [24,25,26,27]. Hence, SWE could hold promise as an objective method for noninvasively evaluating the internal stress of tissues under loading, and could be applied as a tool for quantitatively delineating the mechanical interactions between tissues and clinical interventions in research. In literature, SWE has been applied to investigate the relationships between the mechanical properties of the heel pad and pathologies [28,29,30,31,32,33].

In order to better understand the mechanisms and biomechanical effects of the plastic heel cup for optimizing its design and treatment effects for plantar heel pain, the purpose of the present study was to apply SWE to investigate the effects of the plastic heel cup on the stiffness (which indicates the internal stress, in terms of the shear wave speed) and thickness of MIC and MAC of the heel pad on plantar fasciitis patients during static standing.

## 2. Materials and Methods

### 2.1. Participants

A total of 15 patients (4 men and 11 women; age = 56.9 ± 12.0 years; BMI = 23.2 ± 2.4 kg/m^2^) diagnosed with unilateral plantar fasciitis for more than 1 year were recruited from the outpatient clinic of the department of orthopedic surgery at the National Taiwan University Hospital. The exclusion criteria were neurological diseases with abnormal motor control, as well as trauma or surgical history of foot and ankle. Before testing, each participant was informed of the testing procedures and asked to sign the informed consent approved by the Institutional Review Board of National Taiwan University Hospital.

### 2.2. Experimental Systems and Setups

The Aixplorer ultrasound system (SuperSonic Imagine, Aix-en-Provence, France) equipped with a linear array transducer (SuperLinear SL15-4, 256 elements, center frequency = 8.5 MHz, bandwidth = 4–15 MHz) was used to perform the SWE measurement. The tissue preset and SWE optimization settings of the system were set as general and penetration, respectively. Other settings were set as default. The shear wave speed measured using the system was used as the metric for quantifying the stiffness of tissues since it has been reported that the shear wave speed is directly related to the stiffness of tissues; that is, the higher the shear wave speed, the stiffer the tissue [22,34]. During the SWE measurement, a color map was superimposed on the corresponding B-mode image for displaying the spatial distribution of the magnitude of shear wave speed by colors continuously ranged from red (stiffest) to dark blue (softest). The mean value of the shear wave speed of a local region can be quantitatively measured by a size-adjustable circular region of interest (ROI).

**Figure 3:**
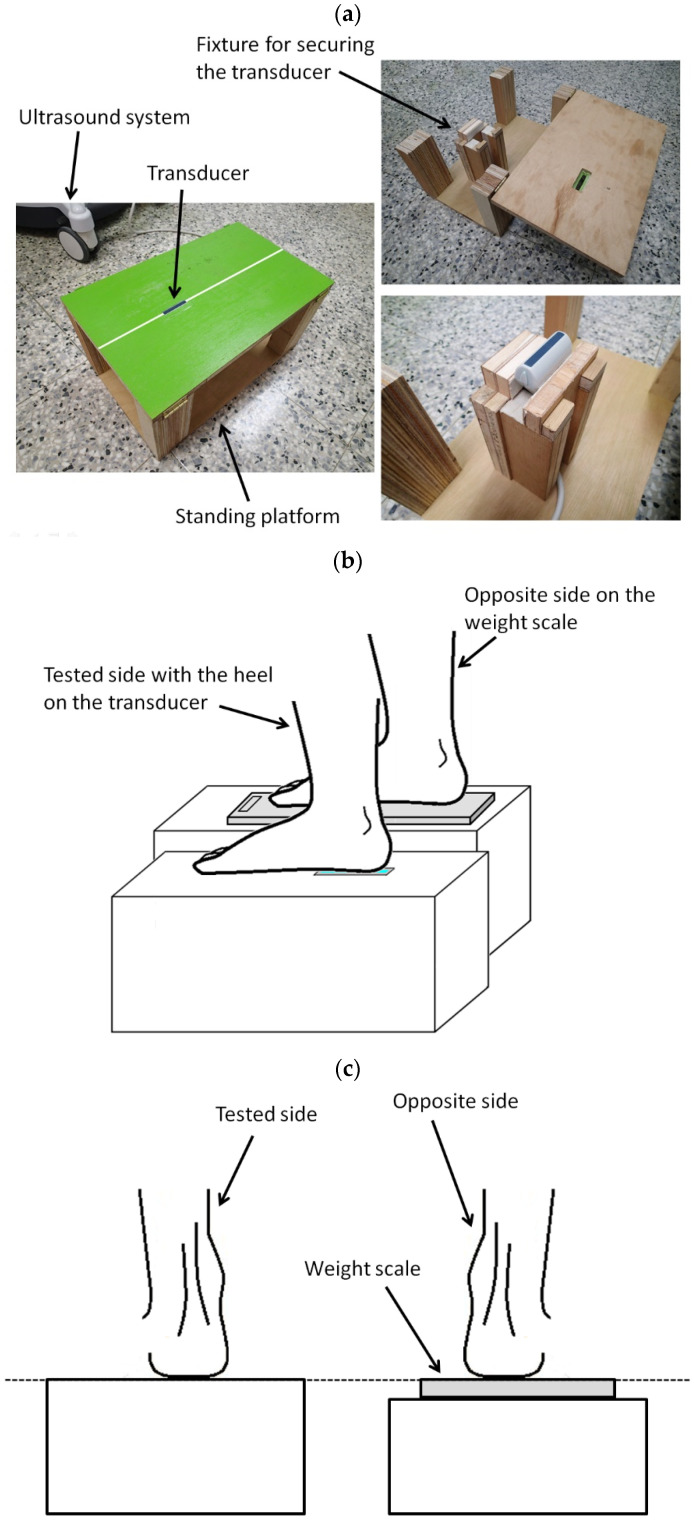
(**a**) The experimental setup for measuring the shear wave speed and thickness of MIC and MAC of the heel pad using SWE during static standing. (**b**) The foot was positioned so that the tested heel was right on the transducer. The other foot was on a weight scale on the other parallel platform. (**c**) The dimensions of both platforms were carefully designed so that there was no height difference between both platforms.

The experimental setup for measuring the shear wave speed and thickness of MIC and MAC of the heel pad using SWE during static standing is illustrated in Figure 3a. The ultrasound transducer was secured by a fixture secured inside the standing platform. The transducer head was embedded in a molded slot so that the surface of the transducer and the surface of the platform were at the same level. Before the measurement, the transducer was applied with an ample amount of ultrasound coupling gel, and then the participant was asked to stand upright on the platform. The foot was carefully positioned so that the tested heel was right on the transducer with the long axis of the foot (the line connecting the second toe and the center of the heel) along the length direction of the transducer (Figure 3b). The other foot was on a weight scale on the other parallel platform (Figure 3b) for ensuring that the weight on each foot was equal and about half of the body weight [35]. The dimensions of both platforms were carefully designed so that there was no height difference between both platforms (Figure 3c).

**Figure 4:**
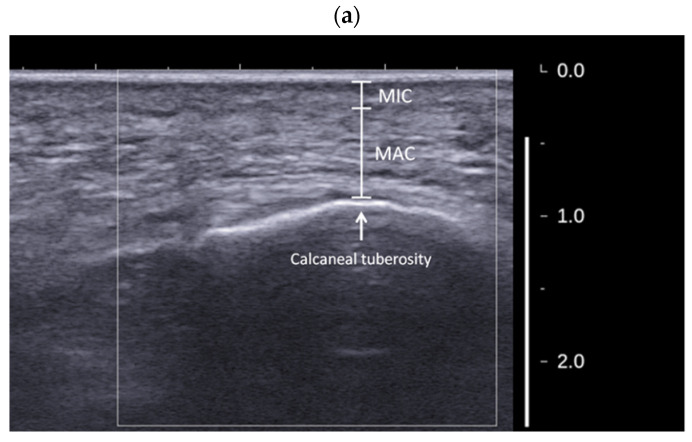

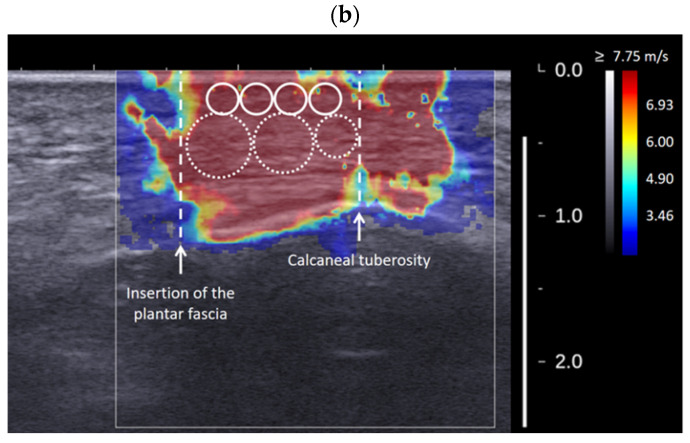
Illustration of the measurement methods for the shear wave speed (**a**) and thickness (**b**) of the microchamber (MIC) and macrochamber (MAC) layers of the heel pad.

During the measurement, B-mode imaging was firstly used to target the heel pad before the SWE measurement. Once a clear image of the heel pad was observed (that is, the calcaneal tuberosity, MIC, and MAC of the heel pad were clearly identified on the image), the SWE mode was activated to measure the shear wave speed. Then, after observing that the color map showing the spatial distribution of the shear wave speed was stable, the image was then captured for further analysis. MIC, MAC, and their thickness values can be clearly identified and measured by B-mode imaging, and please note that the thickness of the skin cannot be included (Figure 4a). To measure the shear wave speed of MIC and MAC, several ROIs (as much as possible) were selected within each layer (Figure 4b). The mean shear wave speed value of each ROI was obtained directly from the SWE system. The shear wave speed of each layer was then defined as the average value of the mean shear wave speed values of all ROIs within each layer. The size of each ROI was adjusted based on the individual anatomy (that is, the thickness of each layer) of each participant. Please note that the plantar fascia should be avoided during the selection of the ROIs for MAC.

It has been reported that, during the SWE measurement using the Aixplorer ultrasound system, there could be a total of three longitudinal band-like artifacts, 1.3 cm apart from each other along the length of the SuperLinear SL15-4 transducer surface, with the middle one at the center of the transducer [36]. If the ROI is mistakenly selected on the region within an artifact, the measurement result is false and cannot reflect accurate information regarding the mechanical properties of tissues. Hence, in order to avoid the artifacts, we had to carefully observe the color map during the measurement and carefully replace the tested heel pad such that the heel pad on the image was in the middle of the region between the artifacts.

### 2.3. Reliability Test of the Measurement Protocol

In order to test the reliability of the measurement protocol, a reliability test group was recruited, consisting of 6 healthy participants (6 men; age = 25.7 ± 3.1 years; BMI = 24.9 ± 2.5 kg/m^2^) without any history of plantar heel pain on both feet. Before testing, each participant was informed of the testing procedures and asked to sign the informed consent approved by the institutional review board of National Taiwan University Hospital. Both heel pads of each participant were tested using the measurement protocol described above, and each heel pad was tested for three successive trials. The heel was repositioned between two successive trials. In each trial, the shear wave speed and thickness of MIC and MAC were measured. The intraclass correlation coefficient ICC (3,1) with 95% confidence interval (CI) was applied to calculate the intra-rater reliability. The reliability test was performed without a plastic heel cup (that is, in barefoot).

The results of the reliability test are described as follows. For MIC, the ICC values for the shear wave speed and thickness were 0.820 (95% CI = 0.607–0.938) and 0.838 (95% CI = 0.595–0.948), respectively. For MAC, the ICC values for the shear wave speed and thickness were 0.845 (95% CI = 0.651–0.948) and 0.981 (95% CI = 0.951–0.994), respectively. These results suggest that the reliability of the measurement protocol was adequate. Please note that this reliability test must be considered as a preliminary test since the number of participants was limited. In addition, only young male participants were recruited. Nevertheless, the reliability of the measurement protocol could be preliminarily verified in this limited number of samples.

### 2.4. Testing Procedure and Analysis Method for Plantar Fasciitis Patients

**Figure 5:**
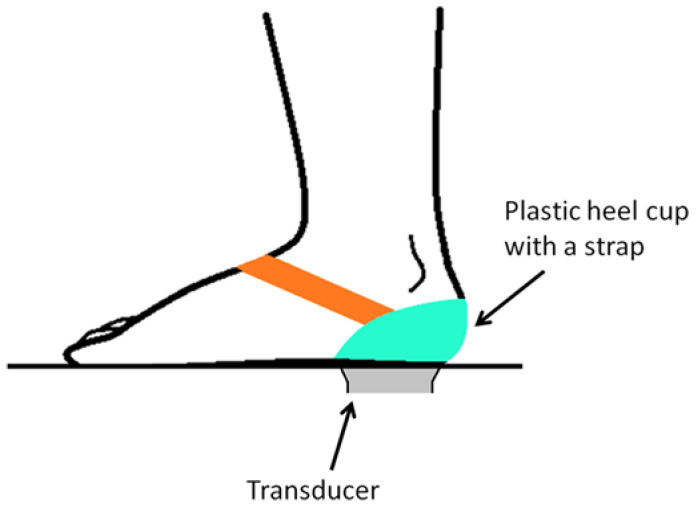
In the measurement with a plastic heel cup, the plastic heel cup was secured to the heel by a strap after applying an ample amount of ultrasound coupling gel to the contact surface of the plastic heel cup and heel.

The shear wave speed and thickness of MIC and MAC of the symptomatic heel pad of each plantar fasciitis patient during static standing were measured with and without plastic heel cup (Innovative Healthcare Ltd., New Taipei City, Taiwan), respectively, using the measurement protocol described above performed by a single tester having more than 5 years of experience in musculoskeletal ultrasound. In the measurement with the plastic heel cup, the plastic heel cup was secured to the heel by a strap after applying an ample amount of ultrasound coupling gel to the contact surface of the plastic heel cup and heel (Figure 5). Each condition (with or without a plastic heel cup) of each heel pad was tested for three successive trials, and the mean value of these three trials was used for the statistical analysis. Paired t-test was applied to determine the difference in the shear wave speed (or thickness) of MIC (or MAC) between the conditions with and without plastic heel cup. The level of statistical significance was set at *p* < 0.05. Statistical analysis and data processing were performed using MATLAB R2021a (The MathWorks, Natick, MA, USA).

## 3. Results

**Figure 6:**
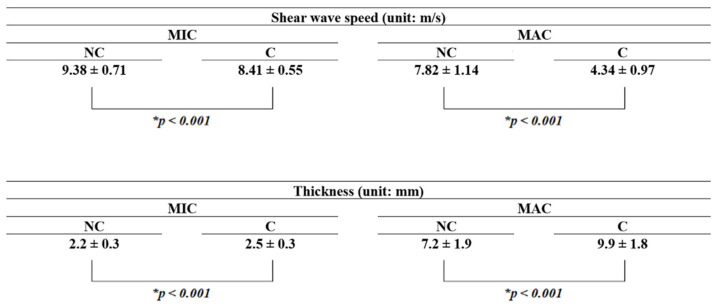
The statistical results. For both the microchamber (MIC) and macrochamber (MAC) layers of the heel pad, the shear wave speed decreased and thickness increased significantly with a plastic heel cup (C) compared with the condition without a plastic heel cup (NC). The asterisk symbol indicates a statistically significant difference.

The results are summarized in Figure 6. For MIC, the shear wave speed values with and without the plastic heel cup were 8.41 ± 0.55 and 9.38 ± 0.71 m/s, respectively (*p* < 0.001; statistical power = 98.56%), while the thickness values with and without the plastic heel cup were 2.5 ± 0.3 and 2.2 ± 0.3 mm, respectively (*p* < 0.001; statistical power = 84.88%), showing that MIC was significantly softer and thicker with the use of the plastic heel cup; these results show that, for MIC, the plastic heel cup helped to reduce the shear wave speed to 89.7% and increase its thickness to 113.6%, compared with the condition without the plastic heel cup. For MAC, the shear wave speed values with and without the plastic heel cup were 4.34 ± 0.97 and 7.82 ± 1.14 m/s, respectively (*p* < 0.001; statistical power = 100%), while the thickness values with and without the plastic heel cup were 9.9 ± 1.8 and 7.2 ± 1.9 mm, respectively (*p* < 0.001; statistical power = 98.64%), showing that MAC is significantly softer and thicker with the use of the plastic heel cup; these results show that, for MAC, the plastic heel cup helped to reduce the shear wave speed to 55.5% and increase its thickness to 137.5%, compared with the condition without the plastic heel cup. In summary, the shear wave speed decreased and the thickness increased significantly with the plastic heel cup for both MIC and MAC, showing that the use of a plastic heel cup can make both layers of the heel cup (and therefore the entire heel cup) significantly softer and thicker. The effect for MAC was more prominent than that for MIC.

**Figure 7:**
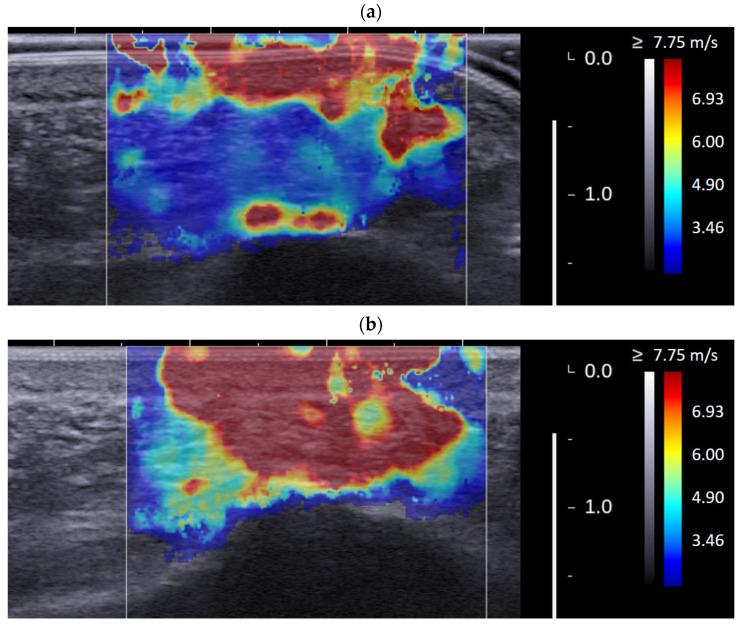
Illustration of an example of the color maps with (**a**) and without (**b**) a plastic heel cup of a symptomatic heel pad of a plantar fasciitis patient. It can be clearly observed that the heel pad (especially for the deep MAC) was significantly softer and thicker with a plastic heel cup.

Figure 7 shows an example of the color maps with and without the plastic heel cup of a symptomatic heel pad of a plantar fasciitis patient. It can be clearly observed that the heel pad (especially for the deep MAC) was significantly softer and thicker with a plastic heel cup, demonstrating that the effects of a plastic heel cup on reducing the stiffness and increasing the thickness of the heel cup are remarkable. Figure 7 just shows an example, but this phenomenon can be observed in each tested heel pad.

## 4. Discussion

The most important finding of the present study is that a plastic heel cup can effectively help to reduce the stiffness (in terms of the shear wave speed) and increase the thickness for both MIC and MAC of the heel pad of the affected side in plantar fasciitis patients during static standing, evaluated by ultrasound SWE. This finding suggests that the mechanism of the plastic heel cup is related to the reduction in the internal stress of the heel pad by increasing its thickness (that is, lowering its deformation) through confinement. It has been reported that the mechanical behaviors of the heel pad are determined by its deformability and mechanical properties [37,38,39], and therefore an alteration in the deformability of the heel pad can change its state of the internal stress, which could explain the finding of the present study. This finding is beneficial for better understanding the mechanisms and biomechanical effects of plastic heel cups, and can be useful for optimizing the design and treatment effects of plastic heel cups for plantar heel pain. To our best knowledge, the present study is the first to investigate the biomechanical effects of the plastic heel cup on the state of the internal stress (in terms of the shear wave speed, which indicates the stiffness) of the heel pad during standing, and we believe that investigating the internal stress of tissues under loading is an important topic to explore because the internal stress is an important mechanical parameter for understanding the mechanisms of pathologies of tissues in the musculoskeletal system. In the present study, we show a new application of SWE, in that SWE can be an objective method for noninvasively evaluating the state of the internal stress of plantar soft tissues during weight-bearing activities. In the future, more studies should be conducted to further investigate the reliability and feasibility of SWE for evaluating the mechanical behaviors of tissues under loading conditions.

The results of the present study show that the effect of a plastic heel cup on MAC is quite considerable, helping to reduce its mean shear wave speed to 55.5% compared with the condition without a plastic heel cup. For MIC, the plastic heel cup helped to reduce its shear wave speed to 89.7% compared with the condition without the plastic heel cup. The degree of the reduction in MIC is lower than that for MAC, probably because MIC is stiffer and thinner in nature. Since MIC is thin and adjacent to the plantar skin, the shear wave speed (that is, the internal stress) of MIC could be positively correlated with the plantar pressure, and the reduction in the shear wave speed of MIC by using a plastic heel cup could imply a reduction in the plantar pressure. Indeed, some previous studies found that the plastic heel cup and similar contoured heel cup inserts can help to reduce plantar heel pressure [10,11,40]. In a finite element computational simulation study, it was found that the effect of a rigid heel cup was to reduce the peak shear stress of the heel pad and plantar skin to 20–42% and 72–78%, respectively [41]. These findings were very similar to those found in the present study. In the future, experimental studies using SWE and plantar pressure sensor simultaneously are needed to further confirm the hypothesis stated above.

MIC and MAC have different functional roles [15]. The functional role of the thicker and softer MAC is to provide cushion and that of the thinner and stiffer MIC is to function as an inherent heel cup to prevent excessive deformation of the deep MAC. The present study found that the shear wave speeds of both layers reduced significantly with the use of a plastic heel cup, suggesting that a plastic heel cup can help to lower the internal stress for both layers. However, the reduction in the shear wave speed of MIC was much lower than that of MAC. Consequently, MIC is still significantly stiffer than MAC during standing with the use of a plastic heel cup, implying that the function of MIC as an inherent heel cup can be well retained.

The causes of plantar heel pain are multiple, including plantar fasciitis, plantar fibromatosis (or called Ledderhose disease), plantar warts, heel pad syndrome, calcaneal stress fracture, and nerve entrapment [42,43]. Heel spurs are often seen in plantar fasciitis patients, but it seems that a heel spur could be merely a phenomenon coexisting with plantar fasciitis but not correlating with the symptoms [42,44,45,46]. In the present study, we recruited patients of plantar heel pain caused specifically by plantar fasciitis as our participants, since plantar fasciitis is considered as the most common cause that leads to plantar heel pain [42,44]. Plantar fasciitis is a pathological degenerative condition of plantar fascia, a connective tissue that connects the plantar heel to the metatarsophalangeal joints [42,43,44]. Plantar fascia plays an important functional role for the biomechanics of foot and locomotion, helping to support the medial longitudinal arch to maintain the foot structure, to dissipate forces during gait to prevent injuries, and to participate in the mechanism of propulsion to enhance locomotion efficiency [44]. Hence, a pathological condition involving plantar fascia such as plantar fasciitis can cause severe adverse effects on the biomechanics of the foot and locomotion. There are several conservative treatment options for plantar fasciitis, including corticosteroid injection, anti-inflammatory medication, extracorporeal shock wave therapy, stretching, night splint, physical therapy, and foot orthoses [4,42,44]. The plastic heel cup investigated in the present study is just one of the treatment options. In an interesting study investigating the anatomy of plantar fascia, it was found that plantar fascia is rich in hyaluronan [44]. Hyaluronan in plantar fascia is produced by fasciacytes, which are fibroblastic-like cells responsible for the production of hyaluronan and fascial gliding regulation [44,47]. Based on this finding demonstrating the richness of hyaluronan in plantar fascia, hyaluronan injection therapy for plantar fascia could be a novel and promising treatment alternative for plantar fasciitis.

There is a major concern in the present study that must be discussed. In the measurement with the plastic heel cup, there was a plastic material between the ultrasound transducer and heel pad, and ultrasound beams had to penetrate through this plastic material before entering the heel pad. However, in the measurement without a plastic heel cup (that is, in barefoot), there was no such plastic material between the transducer and heel pad. In theory, an additional material between the transducer and target tissue might alter the properties of ultrasound transmission, attenuate the energy of ultrasound beams, and therefore cause a decrease in the amplitude of ultrasound beams. However, we believe that this study design could be appropriate since the shear wave speed is dependent on the mechanical properties and density of the tissue but not the amplitude of ultrasound beams [29]. In order to understand whether this study design could be appropriate or not, we performed a preliminary investigation in which we cut a piece of plastic material from a plastic heel cup and put it between the transducer and heel pad of a participant (male; 36 years old; height: 175 cm; weight: 68 kg) during standing, and compared the measured shear wave speeds of both layers to those measured in barefoot. For the left heel pad, the shear wave speeds of MIC and MAC were 7.93 ± 0.12 and 6.97 ± 0.09 m/s, respectively, without the plastic material, and they were 8.03 ± 0.03 and 7.00 ± 0.06 m/s, respectively, with the plastic material. For the right heel pad, the shear wave speeds of the MIC and MAC were 8.07 ± 0.03 and 6.90 ± 0.06 m/s, respectively, without the plastic material, and they were 8.10 ± 0.06 and 6.93 ± 0.07 m/s, respectively, with the plastic material. These results show that the shear wave speeds seem similar under the two conditions without and with the plastic material. Hence, the plastic material between the transducer and heel pad may not affect the measurement of the shear wave speed, and therefore, it could be appropriate to perform the measurement without placing a plastic material between the transducer and heel pad in the condition without the plastic heel cup. Please note that the above finding is only based on one participant and must be considered as a preliminary finding.

There are other limitations in the present study. First, the sample size in the present study (N = 15) is relatively small. Although our results demonstrate that the levels of statistical significance and power are appropriate enough to draw the conclusions even if with a relatively small sample size, we suggest that it is needed to recruit more participants in future studies to test whether the same conclusions can be reached. Second, the commercial one-size plastic heel cup used in the present study could not fit every heel perfectly. The confinement effect could be compromised if the size of the heel of a participant is much smaller than that of the plastic heel cup. However, custom-molded heel cups available in our hospital or commercial market were not suitable for use in the present study because they did not allow transmission of ultrasound beams. Based on the finding of the present study, in order to maximum the confinement and treatment effects, it is suggested that a custom-molded heel cup that perfectly fits the individual size of the heel of a patient should be adopted in the clinical treatment for plantar heel pain. Third, the focus of the present study was to investigate the biomechanical effects of the plastic heel cup; therefore, we did not use a questionnaire to quantify the subjective feelings of patients with the use of the plastic heel cup, and we did not assess the short-term as well as long-term clinical outcomes of plastic heel cups for relieving plantar heel pain. Hence, the relationship between the reduction in the stiffness of the heel pad with the use of a plastic heel cup and the degree of plantar heel pain is still unclear. It is important to investigate this issue in the future in order to confirm the clinical applicability of plastic heel cups. Fourth, the ultrasound elastography system used in the present study can only be applied to static conditions and is not suitable for dynamic activities such as walking because of the relative low frame rate of the SWE acquisition. Hence, the biomechanical effects of the plastic heel cup found in the present study were those during static standing and may not be directly applied to dynamic activities. Nevertheless, it is meaningful to investigate the biomechanical effects of plastic heel cups during standing since some papers reported that prolonged standing not only could trigger and aggravate plantar heel pain [3,48] but also could be a risk factor for plantar heel pain [49,50]. However, understanding of the biomechanical effects of plastic heel cups during dynamic activities is still an important goal in future research. Some technologies can be used to measure the mechanical properties of the heel pad during dynamic activities [11,51], but they can only measure the bulk properties and cannot measure the spatial distribution of the properties within the heel pad; therefore, they might not be able to investigate the respective properties of MIC and MAC. Hence, a novel technology or methodology must be developed for this future research goal.

## 5. Conclusions

In conclusion, by using shear wave ultrasound elastography, we demonstrated that a plastic heel cup can effectively help to reduce the stiffness (in terms of the shear wave speed) and increase the thickness for both microchamber and macrochamber layers of the heel pad during static standing. This finding suggests that the mechanism of a plastic heel cup, and its resulting biomechanical effect, is related to a reduction in the internal stress of the heel pad by increasing its thickness (that is, lowering its deformation) through confinement. This finding is beneficial for better understanding the mechanism and biomechanical effects of plastic heel cups for further optimizing their design and treatment effects for plantar heel pain. Based on the finding of the present study, in order to maximum the confinement and treatment effects, it is suggested that a custom-molded heel cup that perfectly fits the individual size of the heel of a patient should be adopted in the clinical treatment of plantar heel pain.

## Data Availability

The datasets relevant to the present study are available on request to the corresponding author.

## References

[1] Cotchett M.P., Whittaker G., Erbas B. (2015). Psychological variables associated with foot function and foot pain in patients with plantar heel pain. Clin. Rheumatol..

[2] Whittaker G.A., Munteanu S.E., Menz H.B., Elzarka A., Landorf K.B. (2017). Corticosteroid injections compared to foot orthoses for plantar heel pain: Protocol for the SOOTHE heel pain randomised trial. Contemp. Clin. Trials Commun..

[3] McPoil T.G., Martin R.L., Cornwall M.W., Wukich D.K., Irrgang J.J., Godges J.J. (2008). Heel pain—plantar fasciitis. J. Orthop. Sports Phys. Ther..

[4] Rosenbaum A.J., DiPreta J.A., Misener D. (2014). Plantar heel pain. Med. Clin..

[5] Snook G.A., Chrisman O.D. (1972). The management of subcalcaneal pain. Clin. Orthop. Relat. Res..

[6] Montgomery R.M., Locascio W.V. (1966). Padding and devices for foot comfort. Arch. Dermatol..

[7] Scranton P.E., Pedegana L.R., Whitesel J.P. (1982). Gait analysis: Alterations in support phase forces using supportive devices. Am. J. Sports Med..

[8] Katoh Y., Chao E.Y.S., Morrey B.F., Laughman R.K. (1983). Objective technique for evaluating painful heel syndrome and its treatment. Foot Ankle.

[9] Jørgensen U., Ekstrand J. (1988). Significance of heel pad confinement for the shock absorption at heel strike. Int. J. Sports Med..

[10] Wang C.L., Cheng C.K., Tsuang Y.H., Hang Y.S., Liu T.K. (1994). Cushioning effect of heel cups. Clin. Biomech..

[11] Telfer S., Woodburn J., Turner D.E. (2014). Measurement of functional heel pad behaviour in-shoe during gait using orthotic embedded ultrasonography. Gait Posture.

[12] Rome K. (1998). Mechanical properties of the heel pad: Current theory and review of the literature. Foot.

[13] Gefen A. (2003). Plantar soft tissue loading under the medial metatarsals in the standing diabetic foot. Med. Eng. Phys..

[14] Gefen A., Linder-Ganz E. (2004). Diffusion of ulcers in the diabetic foot is promoted by stiffening of plantar muscular tissue under excessive bone compression. Orthopade.

[15] Hsu C.C., Tsai W.C., Wang C.L., Pao S.H., Shau Y.W., Chuan Y.S. (2007). Microchambers and macrochambers in heel pads: Are they functionally different?. J. Appl. Physiol..

[16] Hsu C.C., Tsai W.C., Hsiao T.Y., Tseng F.Y., Shau Y.W., Wang C.L., Lin S.C. (2009). Diabetic effects on microchambers and macrochambers tissue properties in human heel pads. Clin. Biomech..

[17] Hsu C.-C., Chen C.P.-C., Lin S.-C., Tsai W.-C., Liu H.-T., Lin Y.-C., Lee H.-J., Chen W.-P. (2012). Determination of the augmentation effects of hyaluronic acid on different heel structures in amputated lower limbs of diabetic patients using ultrasound elastography. Ultrasound Med. Biol..

[18] Naemi R., Chockalingam N. (2013). Mathematical models to assess foot-ground interaction: An overview. Med. Sci. Sports Exerc..

[19] Teoh J.C., Lee T. (2016). Prediction of plantar soft tissue stiffness based on sex, age, bodyweight, height and body mass index. J. Mech. Behav. Biomed. Mater..

[20] Ahanchian N., Nester C.J., Howard D., Ren L., Parker D. (2017). Estimating the material properties of heel pad sub-layers using inverse Finite Element Analysis. Med. Eng. Phys..

[21] Wu C.H., Lin C.Y., Hsiao M.Y., Cheng Y.H., Chen W.S., Wang T.G. (2018). Altered stiffness of microchamber and macrochamber layers in the aged heel pad: Shear wave ultrasound elastography evaluation. J. Formos. Med. Assoc..

[22] Bercoff J., Tanter M., Fink M. (2004). Supersonic shear imaging: A new technique for soft tissue elasticity mapping. IEEE Trans. Ultrason. Ferroelectr. Freq. Control.

[23] Wells P.N., Liang H.D. (2011). Medical ultrasound: Imaging of soft tissue strain and elasticity. J. R. Soc. Interface.

[24] Eby S.F., Song P., Chen S., Chen Q., Greenleaf J.F., An K.N. (2013). Validation of shear wave elastography in skeletal muscle. J. Biomech..

[25] Koo T.K., Guo J.Y., Cohen J.H., Parker K.J. (2013). Relationship between shear elastic modulus and passive muscle force: An ex-vivo study. J. Biomech..

[26] Zhang Z.J., Fu S.N. (2013). Shear elastic modulus on patellar tendon captured from supersonic shear imaging: Correlation with tangent traction modulus computed from material testing system and test–retest reliability. PLoS ONE.

[27] Martin J.A., Biedrzycki A.H., Lee K.S., DeWall R.J., Brounts S.H., Murphy W.L., Markel M.D., Thelen D.G. (2015). In vivo measures of shear wave speed as a predictor of tendon elasticity and strength. Ultrasound Med. Biol..

[28] Lin C.Y., Lin C.C., Chou Y.C., Chen P.Y., Wang C.L. (2015). Heel pad stiffness in plantar heel pain by shear wave elastography. Ultrasound Med. Biol..

[29] Lin C.Y., Chen P.Y., Shau Y.W., Tai H.C., Wang C.L. (2017). Spatial-dependent mechanical properties of the heel pad by shear wave elastography. J. Biomech..

[30] Taş S., Bek N., Ruhi Onur M., Korkusuz F. (2017). Effects of body mass index on mechanical properties of the plantar fascia and heel pad in asymptomatic participants. Foot Ankle Int..

[31] Taş S. (2018). Effect of gender on mechanical properties of the plantar fascia and heel fat pad. Foot Ankle Spec..

[32] Taş S., Bek N. (2018). Effects of morphological and mechanical properties of plantar fascia and heel pad on balance performance in asymptomatic females. Foot.

[33] Chatzistergos P.E., Behforootan S., Allan D., Naemi R., Chockalingam N. (2018). Shear wave elastography can assess the in-vivo nonlinear mechanical behavior of heel-pad. J. Biomech..

[34] Urban M.W., Nenadic I.Z., Chen S., Greenleaf J.F. (2013). Discrepancies in reporting tissue material properties. J. Ultrasound Med..

[35] Luximon A., Goonetilleke R.S., Zhang M. (2005). 3D foot shape generation from 2D information. Ergonomics.

[36] Lin C.Y., Chen P.Y., Shau Y.W., Wang C.L. (2017). An artifact in supersonic shear wave elastography. Ultrasound Med. Biol..

[37] Naemi R., Chatzistergos P., Sundar L., Chockalingam N., Ramachandran A. (2016). Differences in the mechanical characteristics of plantar soft tissue between ulcerated and non-ulcerated foot. J. Diabetes Complicat..

[38] Naemi R., Chatzistergos P.E., Chockalingam N. (2016). A mathematical method for quantifying in vivo mechanical behaviour of heel pad under dynamic load. Med. Biol. Eng. Comput..

[39] Behforootan S., Chatzistergos P.E., Chockalingam N., Naemi R. (2017). A clinically applicable non-invasive method to quantitatively assess the visco-hyperelastic properties of human heel pad, implications for assessing the risk of mechanical trauma. J. Mech. Behav. Biomed. Mater..

[40] Perhamre S., Lundin F., Klässbo M., Norlin R. (2012). A heel cup improves the function of the heel pad in Sever’s injury: Effects on heel pad thickness, peak pressure and pain. Scand. J. Med. Sci. Sports.

[41] Spears I.R., Miller-Young J.E., Sharma J., Ker R.F., Smith F.W. (2007). The potential influence of the heel counter on internal stress during static standing: A combined finite element and positional MRI investigation. J. Biomech..

[42] Tu P., Bytomski J.R. (2011). Diagnosis of heel pain. Am. Fam. Phys..

[43] Todros S., Biz C., Ruggieri P., Pavan P.G. (2021). Experimental Analysis of Plantar Fascia Mechanical Properties in Subjects with Foot Pathologies. Appl. Sci..

[44] Stecco C., Corradin M., Macchi V., Morra A., Porzionato A., Biz C., De Caro R. (2013). Plantar fascia anatomy and its relationship with A chilles tendon and paratenon. J. Anat..

[45] Onwuanyi O.N. (2000). Calcaneal spurs and plantar heel pad pain. Foot.

[46] Johal K.S., Milner S.A. (2012). Plantar fasciitis and the calcaneal spur: Fact or fiction?. Foot Ankle Surg..

[47] Stecco C., Fede C., Macchi V., Porzionato A., Petrelli L., Biz C., Stern R., De Caro R. (2018). The fasciacytes: A new cell devoted to fascial gliding regulation. Clin. Anat..

[48] Barnes A., Sullivan J., Pappas E., Adams R., Burns J. (2017). Clinical and Functional Characteristics of People with Chronic and Recent-Onset Plantar Heel Pain. PM&R.

[49] Irving D.B., Cook J.L., Menz H.B. (2006). Factors associated with chronic plantar heel pain: A systematic review. J. Sci. Med. Sport.

[50] Landorf K.B. (2015). Plantar heel pain and plantar fasciitis. BMJ Clin. Evid..

[51] Gefen A., Megido-Ravid M., Itzchak Y. (2001). In vivo biomechanical behavior of the human heel pad during the stance phase of gait. J. Biomech..

